# Geographies of asthma medication purchase for pre-schoolers in Belgium

**DOI:** 10.1186/s12931-019-1052-8

**Published:** 2019-05-14

**Authors:** Sonia Trabelsi, Lidia Casas, Benoit Nemery, Tim S. Nawrot, Isabelle Thomas

**Affiliations:** 1 Center for Operations Research and Econometrics, Voie du Roman Pays, 34 bte L1.03.01, B-1348 Louvain-la-Neuve, Belgium; 2 Centre for Environment and Health, KULeuven, Leuven, Belgium; 3 Centre for Environmental Sciences, Hasselt University, Hasselt, Belgium; 4 National Fund for Scientific Research, Brussels, Belgium

## Background

Wheeze is a common symptom among pre-schoolers, with an estimated cumulative prevalence at the age of 6 of up to almost 50% [1, 2]. Wheezing can be a manifestation of many diseases, including respiratory infections and asthma, and it can be triggered by environmental factors such as air pollution, allergens or tobacco smoke [3, 4], some of them very much dependent on the geographical location of the residence. The management of pre-school wheezing is challenging and includes measures to avoid environmental triggers and medication [4, 5]. Nevertheless, evidence on which to base recommendations in pre-schoolers is limited [4] and this results in high numbers of prescriptions [6, 7] and substantial costs (e.g. 0.15% of the healthcare budget in the UK [8]). In addition, the choice of a treatment is influenced by parents or caregivers’ interpretation of the symptoms as well as medical practice by primary care doctors and paediatricians [9, 10], which may be geographically determined.

The concentration of people and activities in large cities leads to many positive effects [11], but also to negative externalities, including socio-economical segregation or high levels of traffic-related air pollution [12]. Children growing up in rural areas may, in contrast, benefit from protective effects on respiratory health due to lower exposure to traffic-related air pollutants, to a more diverse microbial environment or to specific lifestyles (e.g. consumption of unprocessed milk) [13, 14]. However, living in rural areas may also imply poor access to health care [15] and high exposure to other pollutants (e.g. pesticides) [16] or pro-inflammatory agents like endotoxins [17]. In this regard, previous research suggests that the prevalence of pre-school wheezing, the risk of asthma diagnosis, and that of the use of medication for airway-related symptoms may differ between urban and rural environments. While studies comparing urban and rural prevalence of respiratory symptoms in pre-schoolers show a higher prevalence and risk of asthma diagnosis in urban areas [18, 19], the few studies investigating differences in the use of asthma medication show inconsistent results [19, 20]. A potential explanation for these inconsistencies may be the differences in health care access or in medical practice across countries or regions.

Here, we describe the spatial differences in asthma medication prescribed to pre-schoolers within Belgium, and evaluate the correlations with some relevant socio-economic and environmental factors, such as population density, income, air pollution, farming activity and greenness.

## Materials and methods

### Studied area

Belgium is a small but densely inhabited European country (11.35 million inhabitants over 30,528 km^2^), administrated federally into three Regions: the Flemish, the Walloon and the Brussels Capital Region (see Additional file 3: Figure S1). Belgium is further divided administratively into 10 provinces, 43 “arrondissements” and 589 municipalities (see Additional file 1: Table S1). Recently, medication reimbursement data have become available at different statistical levels: provinces, municipalities, statistical sections and statistical sectors [21], enabling to study spatial variations at different levels of aggregation. The smallest statistical sector is 0.01 km^2^ large and the largest one is over 6.3 km^2^; 24% of the statistical sectors contain less than 100 inhabitants.

The three Regions differ in language, as well as culture, traditions, living habits, historical, or socio-economic conditions. Although social security and health care are federal matters governed at the scale of the entire country, education (including higher education) is a “community” matter [22] and, thus, governed regionally. Except for Brussels Capital Region, mobility of workers between regions in Belgium is limited [23] and only very few medical doctors practice in another Region than the one where they obtained their degree. Also, very little communication takes place between medical practitioners across the regional (linguistic) divide. It is also important to note that, for historical and geographical reasons the Belgian city network is very tight in the North, leading to high densities of population and activities and, consequently, to a high level of air pollution [24], while in the South the city network is - on the average - looser, inter-city distances are much larger and landscapes more hilly and green. In most Belgian cities, better-off people tend to live in the peripheries, and poor people in city centres or in former industrial areas [25–28].

We analysed the geographical distribution of prescriptions of asthma medication to pre-schoolers at three levels, with increasing degrees of spatial resolution: first, the whole of Belgium, using its 589 municipalities as spatial units; then, the former province of Brabant, using 375 former municipalities (as they existed before being merged in 1977) as spatial units; and, finally, the Brussels Capital Region (BCR), using its 147 statistical sections as spatial units.

The former province of Brabant is the central area of Belgium that contains, in its centre, the Brussels Capital Region (BCR), a politically defined entity (161 km^2^ and 1.2, Mio inhabitants), surrounded by “Vlaams-Brabant” (2106 km^2^, 1.1 Mio inhabitants, Flemish speaking) and “Brabant Wallon” (1090 km^2^, 0.4 Mio inhabitants, French speaking). Whereas the BCR is fully urban, both Flemish and Walloon Brabant contain urban, suburban and some rural areas.

### Medication purchases

Almost all Belgian residents (98%) are enrolled in the social security system. The information on individual health care expenditures that are reimbursed by the seven “sickness funds” is centralized by a single agency [Intermutualistisch Agentschap – Agence Inter-Mutualiste (IMA-AIM)]. The agency’s database contains detailed records of all reimbursed drugs by ATC code (Anatomical Therapeutic Chemical [29]) over defined calendar periods, for each patient identified by national security number, age, gender and place of residence. The use of these data is governed by the principles of the Declaration of Helsinki. For the present study, we obtained data aggregated by administrative spatial units (provinces, current municipalities, statistical sections and statistical sectors) on medication, reimbursed in 2014, included in the R03 ATC code (i.e. “*Drugs for obstructive airway diseases*”, [30]), with the limitation of not having any information on the exact medication type or the frequency of use.

We limited our analyses to children aged 0 to 5, named here pre-schoolers. Our database did not allow us to get finer age groups, but it was possible to compare pre-schoolers to older children: 6–12 years and 13–18 years. The diagnosis of asthma can indeed be established more firmly in children above 6 years, but because wheezing is less frequent among primary schoolchildren and adolescents, the numbers of individuals receiving asthma medication are much lower than among pre-school children, thus leading to statistical representativeness problems, especially in small spatial units. Nevertheless, the geographical distribution of reimbursements for asthma medications for schoolchildren (6-12y) and adolescents (13-18y) exhibited the same spatial structures (not illustrated here), and similar but statistically less representative results were obtained for the Pearson correlations between the prevalence of asthma medication reimbursements and the environmental variables described for pre-schoolers (0-5y) in Table 1.

**Table 1:** Correlation coefficients of prevalence of purchase and cost/case and explanatory variables

	Prevalence of purchase	Cost/case (log)
Population density (log)	−0.587	− 0.057 (n.s.)
Median Income/declaration	−0.230	0.034 (n.s.)
PM_10_ (μg/m^3^)	−0.444	0.172
PM_10_ P95 (μg/m^3^)	−0.470	0.221
density of pigs (log)	0.379	0.328
% Green	0.544	0.145

The table reports Pearson correlation coefficients between prevalence of purchase of asthma medication for pre-school children (left column) or cost/case (right column), and selected explanatory variables in municipalities in Belgium (significant - *α =* 0.001; *n.s.* Not significant; 588 municipalities)

The data used here are: (1) the number of *cases* in an administrative spatial unit (municipality, former municipality, statistical section), in other words, the number of pre-schoolers aged 0–5 years that benefitted from reimbursement at least once in 2014 for any purchase of a prescribed drug with an R03 ATC code, (2) the total number of pre-schoolers registered in the social security system in 2014 in the administrative spatial unit, and (3) the total *cost* (reimbursed + out-of-pocket contribution) of the medications reimbursed in 2014 in the administrative spatial unit. It is worth noting that in Belgium the price of the medications is fixed at the federal level [31], and prices are, therefore, identical in all three Regions. For privacy reasons, IMA-AIM does not provide the exact address of the patients but only the code of the administrative spatial unit of his/her current declared residence, and information is not provided if less than 5 cases of registered pre-schoolers are recorded in a spatial unit.

The three variables described above ((1) number of cases, (2) number of registered pre-schoolers, and (3) total costs of reimbursed medication) were then further combined into two relative variables: the prevalence of purchase (in %) and the costs per case (in €). The *prevalence* of purchase was computed as the number of cases (1) divided by the number of registered pre-schoolers (2) and multiplied by 100. To further explore potential geographical differences in the frequency of medication use, we also computed the *costs per case* for each administrative spatial unit. This is the total costs for reimbursed asthma medication (3) divided by the number of cases (1); it is interpreted as follows: the higher the costs per case, the higher the probability of having chronic users in an administrative unit. Thus, two areas with the same prevalence may be characterised by completely different consumption profiles: sporadic users (low cost per case) and chronic users (high cost per case).

### Characterising the environment

Data about *green environment* were derived from the Corine Land Cover dataset, a European land use dataset, developed by the European Environment Agency [32]. Four typologies of green spaces were initially considered (urban green, pastures and agricultural land, forests, and semi-natural areas) and further summed up. The percentage of the total surface occupied by these green spaces in each spatial unit was used as a measure of greenness.

Data about *air pollution* were provided by the Belgian Interregional Environment Agency, as daily average Particulate Matter 10 (PM_10_) values estimated for areas of 4 × 4 km. These estimates are based on a spatial-temporal (Kriging) interpolation model that combines pollution data collected on a half-hourly basis by a dense network of automatic monitoring stations and satellite-based land cover images [33]. These data were used to calculate area-weighted average concentrations of PM_10_ per spatial unit. The value of the 95th percentile (noted PM_10_P95) of the yearly average of PM_10_ per municipality was also considered.

The 2014 data on population density and median income at different spatial levels, as well as the information on the density of pigs, were obtained from the Belgian statistical office (Statistics Belgium).

## Methodology

Data were mapped using two different methods: first, a proportional symbol map, where the surface of the circles is proportional to the number of reimbursements. This type of maps gives an idea of the spatial variation of the absolute numbers. Secondly, we used choropleth maps where spatial units are shaded according to prevalence, i.e. the relative numbers of medication reimbursements; the limits of the classes of values are fixed by a natural breaks classification [34]. The latter minimises intra-class variation and maximises inter-class variation. However, for the cross-scale analysis (Additional file 4: Figure S2) another discretisation method was used to enable comparisons between maps: the standard deviation method [34].

To examine the relationships of prevalence of purchase and costs per case with socio-economic and environmental variables at the level of the municipality, we calculated Pearson correlations.

Although data were available for all municipalities, considering smaller spatial units, such as former municipalities and statistical sections, automatically lead to missing data problems (privacy and statistical issues) and the non-availability of some variables for very small units. Spatial units with missing data were removed from the analysis.

## Results

Basic descriptive statistics about prevalence of purchase, cost per case and environmental and socio-economic variables at the level of the Belgian municipalities are reported in Additional file 2: Table S2.

### Geographic variation at the country level

**Fig. 1:**
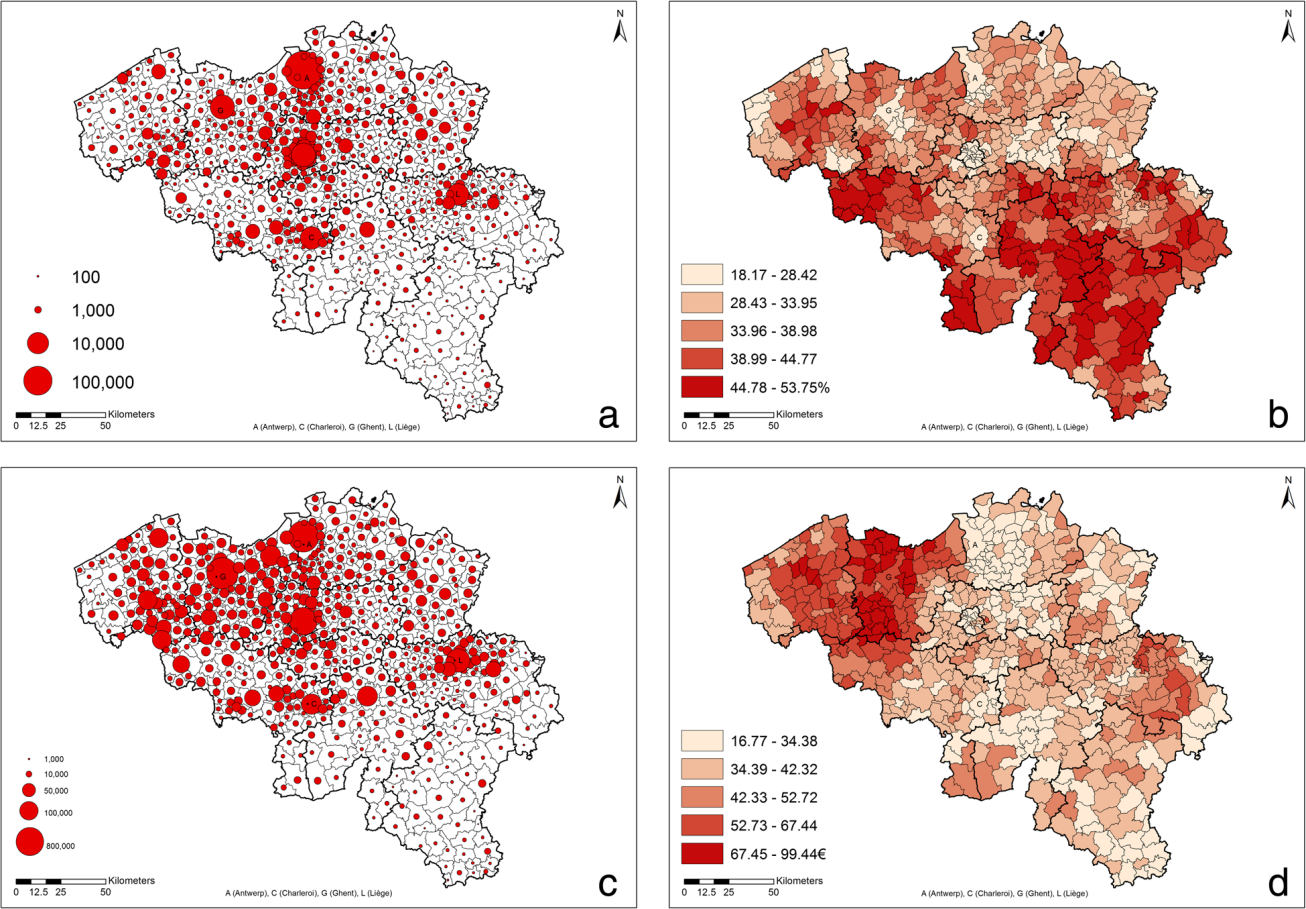
Asthma medication purchases for pre-schoolers by municipalities: **a** Number of 0-5y children reimbursed at least once in 2014 for asthma medication (for comparability reasons the 19 municipalities of Brussels are summarised into 1 entity); **b** Prevalence of purchase of asthma medication among 0-5y children in 2014; **c** Total cost per municipality for asthma medication for 0-5y children; **d** Cost/case per municipality for asthma medication for 0-5y children

The spatial distribution of the absolute numbers of pre-school age children that benefitted from at least one prescription of asthma medication in 2014 (ATC code R03) is shown in Fig. 1a. Numbers vary from a minimum of 78 to a maximum of 44,144, with an average value of 1281, and a median value of 744. The map clearly reflects the tight urban network of the northern part of the country and the less populated southern part of the country. Also the big cities (Antwerp, Ghent, Brussels, Charleroi and Liège), clearly appear.

In contrast, the map of the prevalence of purchase of asthma medication (Fig. 1b) showed low values in large cities (minimum of 18%), whereas the highest values were observed mainly in Wallonia (with a maximum of 54%), evidencing the country’s North-South division with an average prevalence of 33% (SD = 5.5) in the Flemish region, and 41% (SD = 6) in the Walloon region.

As for the absolute number of cases, total costs (Fig. 1c) were high in urbanized municipalities, with a maximum expenditure of 291,825€ (and a minimum of 735€); on average the cost per municipality was 17,438€ (median 11,332€). However, costs were also much higher in the North Western part of the country. This structure persisted and was even stronger when considering the relative expression: cost/case (Fig. 1d). In Belgium, high values of the cost per case were concentrated in areas where the prevalence of purchase (Fig. 1b) was relatively small.

Table 1 provides pairwise Pearson correlation coefficients between prevalence of purchase and cost/case on the one hand, and population density, income and the selected environmental factors, on the other hand. Confirming the visual appraisal (Fig. 1b), prevalence of purchase was negatively correlated with population density and, as a consequence, positively related with the percentage of green; prevalence was negatively correlated with income and particulate air pollution. In other words, green areas far away from urban and polluted areas were characterised by a higher prevalence of purchase, confirming the results showed in the maps. In contrast, cost/case was poorly correlated with most variables but the correlation with PM_10_ was positive.

### Zooming into Brabant and the Brussels capital region

**Fig. 2:**
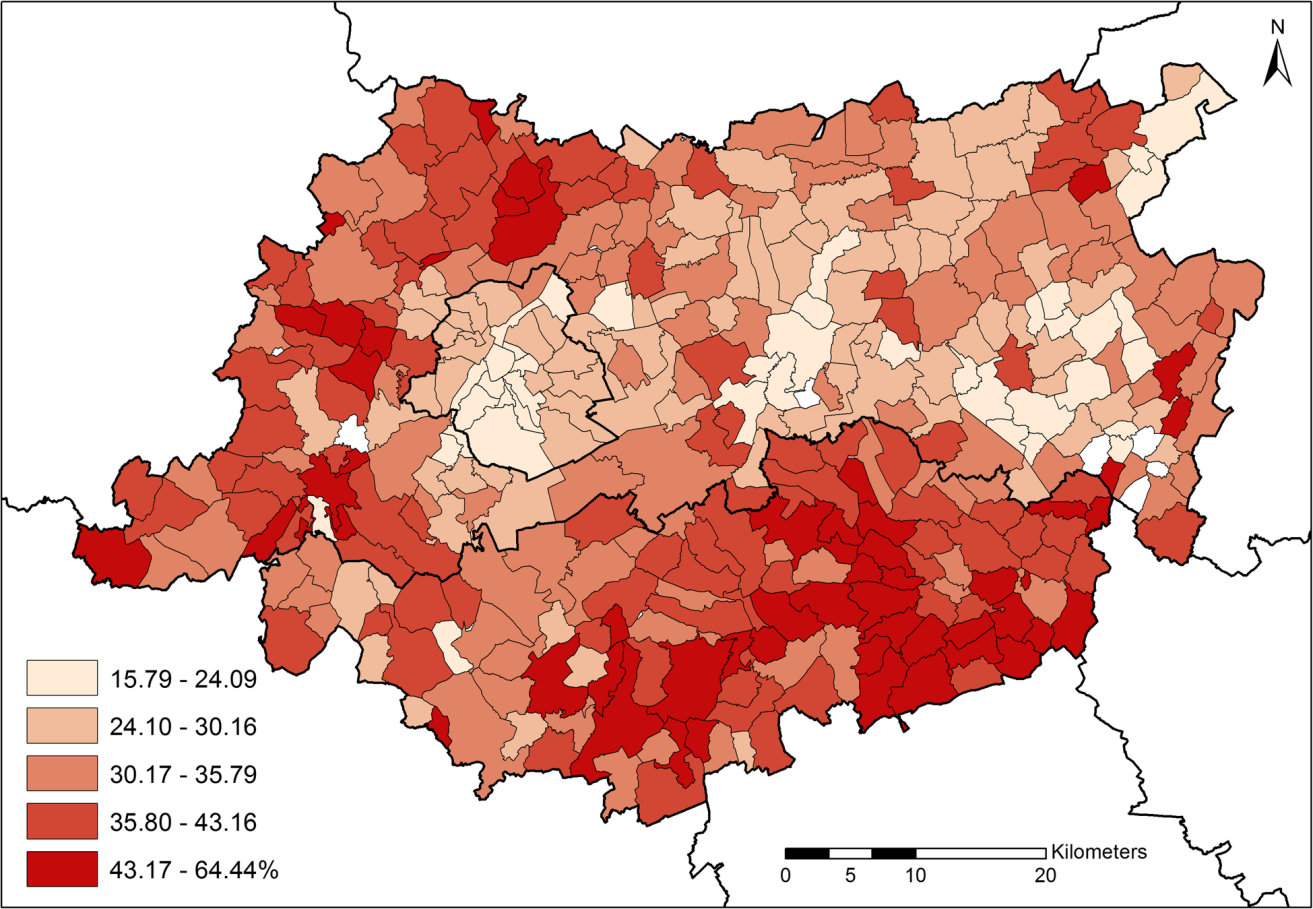
Prevalence of purchase of asthma medication in the former Province of Brabant among 0–5 y children (2014)

**Fig. 3:**
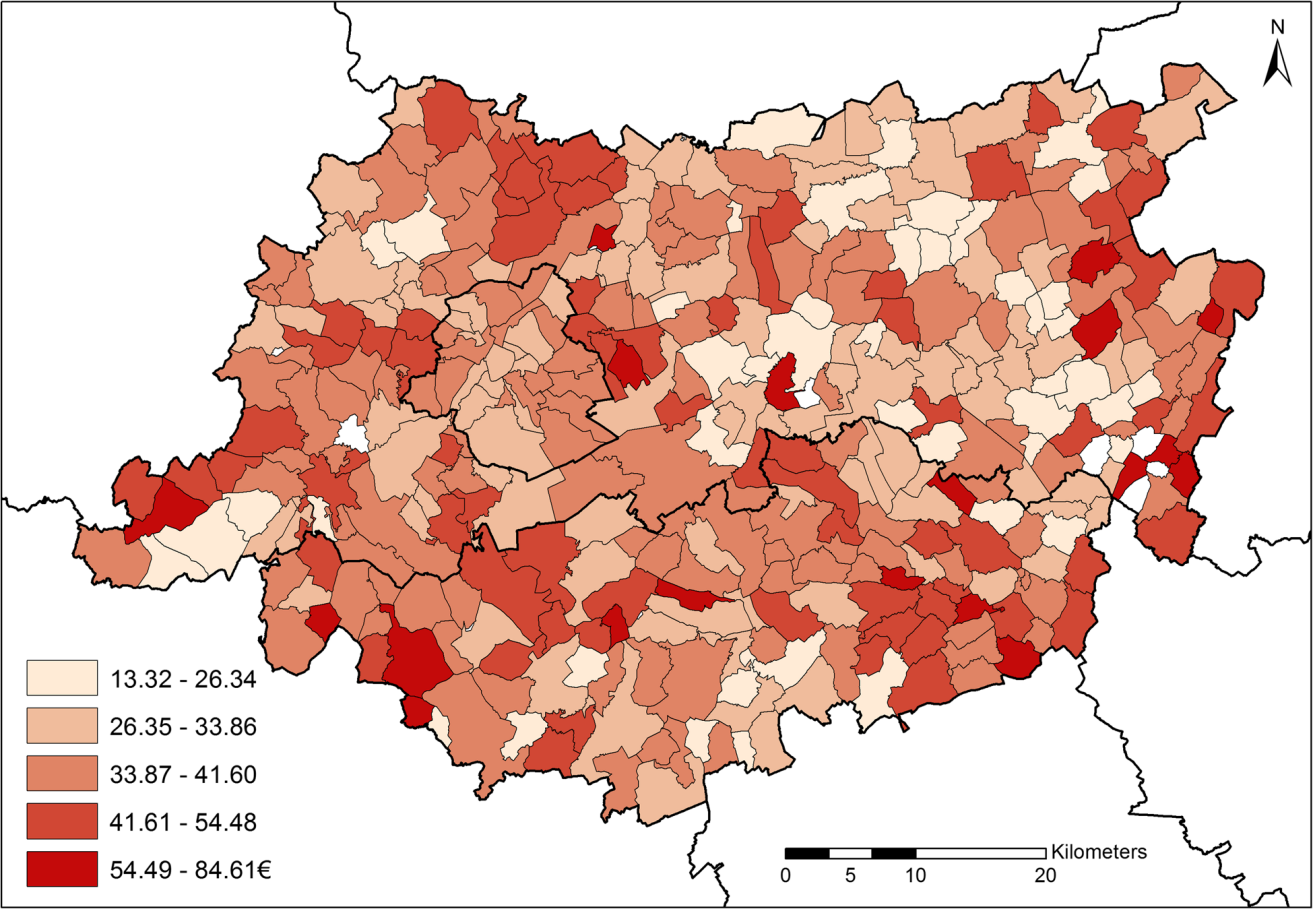
Cost/case of asthma medication in the former Province of Brabant among 0–5y children (2014)

Zooming into the former province of Brabant, the values of prevalence of purchase ranged between 16 and 64%, with an average of 34% (Table 2). Spatially, prevalence of purchase was higher outside the BCR (Fig. 2), and mainly concentrated in the Eastern part of Brabant Wallon and in the Western part of Vlaams-Brabant. In contrast, the cost/case distribution (Fig. 3) appeared to occur at random without any spatial autocorrelation of the values.

**Table 2:** Descriptive statistics of prevalence of purchase and cost/case for the three studied areas

	Spatial unit			Prevalence of purchase	Costs per case
	Administrative level	Total #	Retained #	%	€
Belgium	Municipalities	589	588	36.40 (7.01)	42.72 (13.05)
Brabant	Former municipalities	375	357	33.62 (8.38)	36.55 (11.15)
BCR	Statistical sections	147	141	24.60 (3.63)	34.32 (6.52)

The table reports descriptive statistics of prevalence of purchase and cost/case for asthma medication in the three nested studied areas (where “Retained #” is the number of spatial units for which data were not missing)

**Fig. 4:**
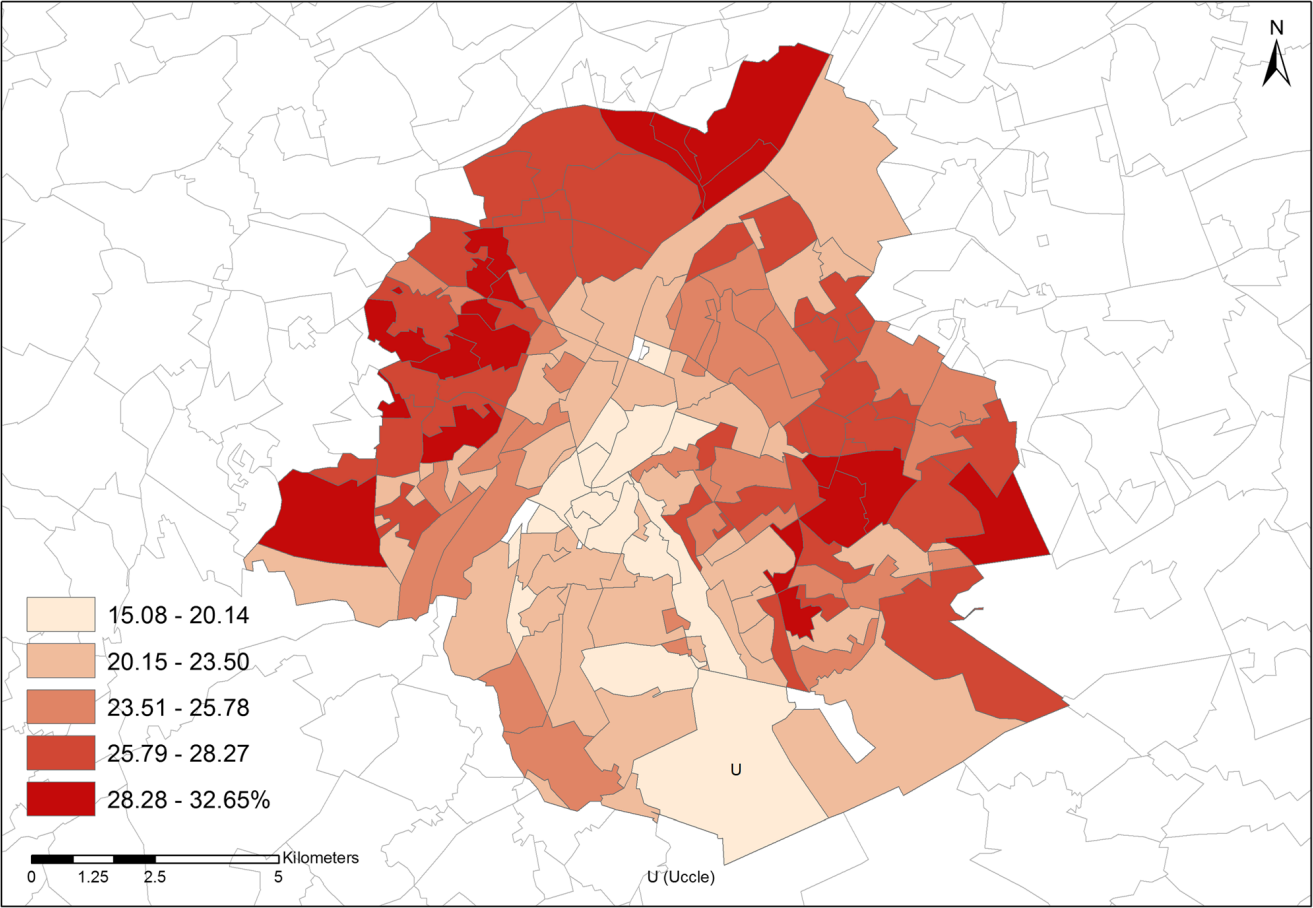
Prevalence of purchase of asthma medication among 0–5y children by statistical sections within the Brussels Capital Region (2014)

Figure 4 shows the intra-urban variation of prevalence of purchase at the level of the sections within the BCR, with a strong spatial autocorrelation observed in its Western and Eastern parts. Cost/case (not illustrated here) again varied randomly. The municipality of Uccle (the largest municipality in the S-E of the BCR) was conspicuous for its low values. The average value was much lower at the level of Brussels compared to the two Provinces (see Table 2).

Additional file 4: Figure S2 presents the variation in prevalence of purchase for the three studied areas, but with a discretisation method based on standard deviations, allowing better comparisons between the three different scales.

## Discussion

We described the geographical distribution of asthma medication purchases for pre-schoolers in Belgium. The prevalence of purchase ranged between 18 and 54% in 2014, with higher values observed in the southern part of Belgium, where population density and air pollution concentrations are low, and the amount of green spaces is the highest. In contrast, the costs per case, considered as a proxy for frequency of use, were higher in the north-western part of the country. To our knowledge, no previous publication has mapped such prescription/sales of asthma medication. Only one study investigated the trends and geographical distribution of hospital admissions due to paediatric asthma or asthma-like symptoms in France [35]. The authors observed the highest rates in the North of the country. Although they hypothesized about environmental exposures, primary care attention, or socio-economic conditions as potential explanatory factors, they did not investigate potential correlations.

We calculated correlations with available environmental and socio-economic variables intending to understand the observed geographical distribution of asthma medication purchase. We found inverse correlations between prevalence of purchase and income as well as PM_10_ concentrations, and positive but moderate correlations with green spaces and density of pigs, considered as a proxy for a well-known type of polluting farming activity. Our results are consistent with previous research showing a high prevalence of wheezing in the lowest socio-economic groups [36, 37]. Recent studies on green spaces and asthma-like symptoms showed inconsistent results, suggesting that this relationship is complex, with the concept of green spaces combining a series of protective factors (e.g. low air pollution) and risk factors (e.g. allergens) [17, 36–39]. In our study, we did not have detailed enough information to disentangle this. Finally, the positive correlation we observed with farming activities is consistent with previous research conducted in the Netherlands where the number of livestock within a 1000 m radius explained 12% of the variation in endotoxin levels in the air, a pro-inflammatory component of the bacterial wall [17]. Although living close to or in farms [14, 40] during childhood may protect against the development of asthma, we should keep in mind that only a small proportion of pre-schoolers receiving asthma medication are or will be confidently diagnosed with asthma [1].

So far, only two studies have specifically looked at the geography of asthma medication use and described urban vs rural differences in asthma symptoms and medication use in children. Although both studies showed higher prevalence of symptoms in urban children, their results for medication use are inconsistent [19, 20]. In Portugal, asthmatic children (0–17 years old) from rural areas were less likely to report using any inhaled therapy than children from urban areas [20]. In Bavaria (Germany), asthma medication intake among pre-schoolers was significantly higher in rural compared with urban areas [19]. Our results regarding prevalence of purchase are consistent with those shown in the Bavarian study. A potential explanation for this finding is related to health care access, as distances to health centres or general practitioners are larger in less densely populated areas [41].

However, another potential explanation for the high prevalence of purchases in less densely populated areas in Belgium is the medical practice. Previous research showed that despite the availability of international guidelines for the management of asthma, prescribing practices vary considerably across countries [42]. The Belgian primary health care system is mostly based on small single-handed practices. Although international clinical guidelines are available, they are not compulsory and, therefore, they are not well followed. Moreover, guidelines may vary by linguistic region, with Flemish and French-speaking doctors being influenced by recommendations and practice in The Netherlands and France, respectively [43]. Since the evidence on which to base recommendations in pre-schoolers is limited [4], the “school of medicine” may play a role in the management of asthma-like symptoms in this age group. In this regard, a sharp regional (and linguistic) border is clearly apparent on our maps of the prevalence of purchase. Differences in prescription of medication between the two main linguistic communities in Belgium have not been published, however a study showing differences between Flemish-speaking and French-speaking regions in euthanasia practice in Belgium suggests that – admittedly, ill-defined – cultural factors do play a role in medical practice [44].

The distribution of costs per case did not follow the same pattern as that of prevalence. The costs per case were weakly but positively correlated with PM_10_ concentrations and green spaces and moderately with pig density. Geographically, the area with high costs per case corresponds to the course of a major river (Scheldt) in the north-west, an area characterised by a slightly milder climate, and a high number of days with extreme PM_10_ and PM_2.5_ pollutions [24]. In addition, the difference in the distribution of prevalence and costs per case may be related to the definition of both variables. While prevalence may be a proxy for both recurrent wheezing and children with only one wheezing episode in 2014, an area with high costs per case can be interpreted as an area with more recurrent wheezing, thus pointing with more confidence to an environmental explanation.

This study relied on information about the medications that are reimbursed by the sickness funds for every person covered by the Belgian social security system. Reimbursement data cover a broader range of disease severity compared with hospital admissions, emergency room visits or mortality [45]. However, some limitations need to be acknowledged. First, a small part (2%) of the Belgian population is not covered by the social security and, therefore, not included the statistics (i.e. mainly: undocumented immigrants, children of people employed by international bodies - EU, NATO, etc.). Second, the IMA-AIM agency does not provide information if there are less than 5 cases in a spatial unit (privacy issue). For R03 medication in pre-schoolers, this was the case for only one out of 589 municipalities in Belgium, eighteen out of 375 former municipalities in Brabant, and six out of 147 sections in the BCR. Third, we did not have information on asthma medication at individual level, nor for smaller age categories with the group of “pre-schoolers” (1 year for instance). We know that one third of the infants has at least one wheezing episode by the age of 1 [2], and a second peak in asthma medication use is observed at the age of 4 [6]. Taking here a 5-years-large group hence smoothens these effects. Analysis for R03 medication reimbursement for older children yielded a structurally similar spatial pattern with regard to the prevalence of purchase, including the striking north-south divide observed at national level. Last but not least, drug purchase does not necessarily equate drug use. Also, we were not able to consider the proportions of specific types of medication included in the R03 code (e.g. short-acting beta agonists, long-acting beta agonists, inhaled corticosteroids,…) which would have helped distinguishing children with recurrent wheezing and potential asthma from those having sporadic wheezing more likely related with a respiratory infection. Finally, although the costs of specific medications do not differ between regions, they do vary between types of medications and between generic vs non-generic types, which could be prescribed differently by region. Unfortunately, we also were not able to document these points with the provided data.

The ecological design of our study entails the possibility of ecological fallacy. We did not have any detail about the season of the treatment, the exact reason of the prescription, or the individual clinical record. In addition, although we did correct for socio-economic status at the administrative level, we did not know the individual’s/household’s socio-economic characteristics, lifestyle, exposure to pollutants, or distance to the closest healthcare centre. An in-depth individual survey would be necessary to further investigate the determinants of the distribution of asthma medication purchases. Nevertheless, as previously mentioned, the correlations between the studied health outcomes and potential determinants are supported by the results of previous individual studies, thus reducing the risk of presenting an ecological fallacy.

## Conclusion

We showed that, in Belgium, the geographical distribution of purchases of prescribed asthma medication for pre-schoolers was only weakly correlated with some quite commonly used environmental factors. The fact that we observed clear differences in prevalence of purchase between Flemish and French-speaking regions, combined with the fact that evidence for recommendations in the management of pre-school wheezing is limited, suggests that the observed geographical distribution may be driven by differences in medical/pharmaceutical practices, rather than by environmental realities, and that medical purchases can hardly be considered as a good proxy for health.

## Additional files

Additional file 1: **Table S1.** List of Belgian statistical levels, by size, in decreasing order (DOCX 12.2 kb)

Additional file 2: **Table S2.** Description and statistics of the variables employed in the study (DOCX 13.7 kb)

Additional file 3: **Figure S1.** Administrative partition of Belgium. Provinces. West-Vlaanderen, Oost-Vlaanderen, Antwerpen, Vlaams-Brabant (containing the Brussels Capital Region), Limburg, in Flanders; Hainaut, Brabant Wallon, Liège, Namur, Luxembourg, in Wallonia (PNG 139 kb)

Additional file 4: **Figure S2.** Prevalence of purchase for the three studied areas by means of the discretisation method based on average value and standard deviation (PDF 664 kb)

## Abbreviations

ATC: Anatomical Therapeutic Chemical
BCR: Brussels Capital Region
EU: European Union
IMA-AIM: Intermutualistisch Agentschap – Agence Inter-Mutualiste
NATO: North Atlantic Treaty Organization
PM_10_: Particulate Matter 10
PM_10_P95: 95th Percentile of PM_10_
PM_25_: Particulate Matter 2.5
R03: Drugs for obstructive airway diseases
