# Supplementary material for: Geographies of asthma medication purchase for pre-schoolers in Belgium
Source: Respir Res. 2019 May 14;20:90. doi: 10.1186/s12931-019-1052-8 (PMC6518669; doi:10.1186/s12931-019-1052-8)
Supplement: Supplementary file 1 — Table S1. List of Belgian statistical levels, by size, in decreasing order (DOCX 12.2 kb) [file 12931_2019_1052_MOESM1_ESM.docx]

**Table S1** List of Belgian statistical levels, by size, in decreasing order

| Belgian administrative/spatial units | |
| --- | --- |
| 3 | Regions (Flanders, Wallonia, Brussels) |
| 10 | Provinces |
| 43 | Arrondissements |
| 589 | Municipalities* |
| 2739 | Former municipalities (until 1977)* |
| 6344 | Statistical sections* |
| 19782 | Statistical sectors |

(*) Levels used in this paper
