# Supplementary material for: Geographies of asthma medication purchase for pre-schoolers in Belgium
Source: Respir Res. 2019 May 14;20:90. doi: 10.1186/s12931-019-1052-8 (PMC6518669; doi:10.1186/s12931-019-1052-8)
Supplement: Supplementary file 2 — Table S2. Description and statistics of the variables employed in the study (DOCX 13.7 kb) [file 12931_2019_1052_MOESM2_ESM.docx]

**Table S2** Description and statistics of the variables employed in the study

|  | **Units** | **Mean** | **Std dev** | **Min** | **Max** | **Median** | **95^th^ Percentile** | |
| --- | --- | --- | --- | --- | --- | --- | --- | --- |
| **Prevalence of purchase** | % | 36.40 | 7.01 | 18.17 | 53.75 | 36.21 | 48.71 |  |
| **Cost/case/year** | Euros (€) | 42.72 | 13.15 | 16.77 | 99.44 | 38.53 | 70.22 |  |
| **Population density** | Inhab./km^2^ | 767.78 | 2134.82 | 24.85 | 24027.9 | 305.89 | 1972.97 |  |
| **Income/declaration** | Euros (€) | 24967 | 2721.93 | 14998 | 34229 | 25386 | 28744.4 |  |
| **PM_10_** | µg/m^3^ | 16.47 | 2.96 | 5.81 | 22.55 | 17.23 | 20.4 |  |
| **PM_10_ P95** | µg/m^3^ | 37.51 | 5.21 | 18.7 | 48 | 38.8 | 44.46 |  |
| **Density of pigs** | Pigs/km^2^ | 74.29 | 57.14 | 0 | 471.17 | 64.34 | 175.76 |  |
| **% Total Green** | % land | 72.56 | 19.68 | 0 | 98.54 | 76.8 | 95.36 |  |

Statistics have been computed based on all Belgian municipalities, with the exception of Herstappe (final *n*=588).
