# Supplementary figures and images for: Geographies of asthma medication purchase for pre-schoolers in Belgium
Source: Respir Res. 2019 May 14;20:90. doi: 10.1186/s12931-019-1052-8 (PMC6518669; doi:10.1186/s12931-019-1052-8)

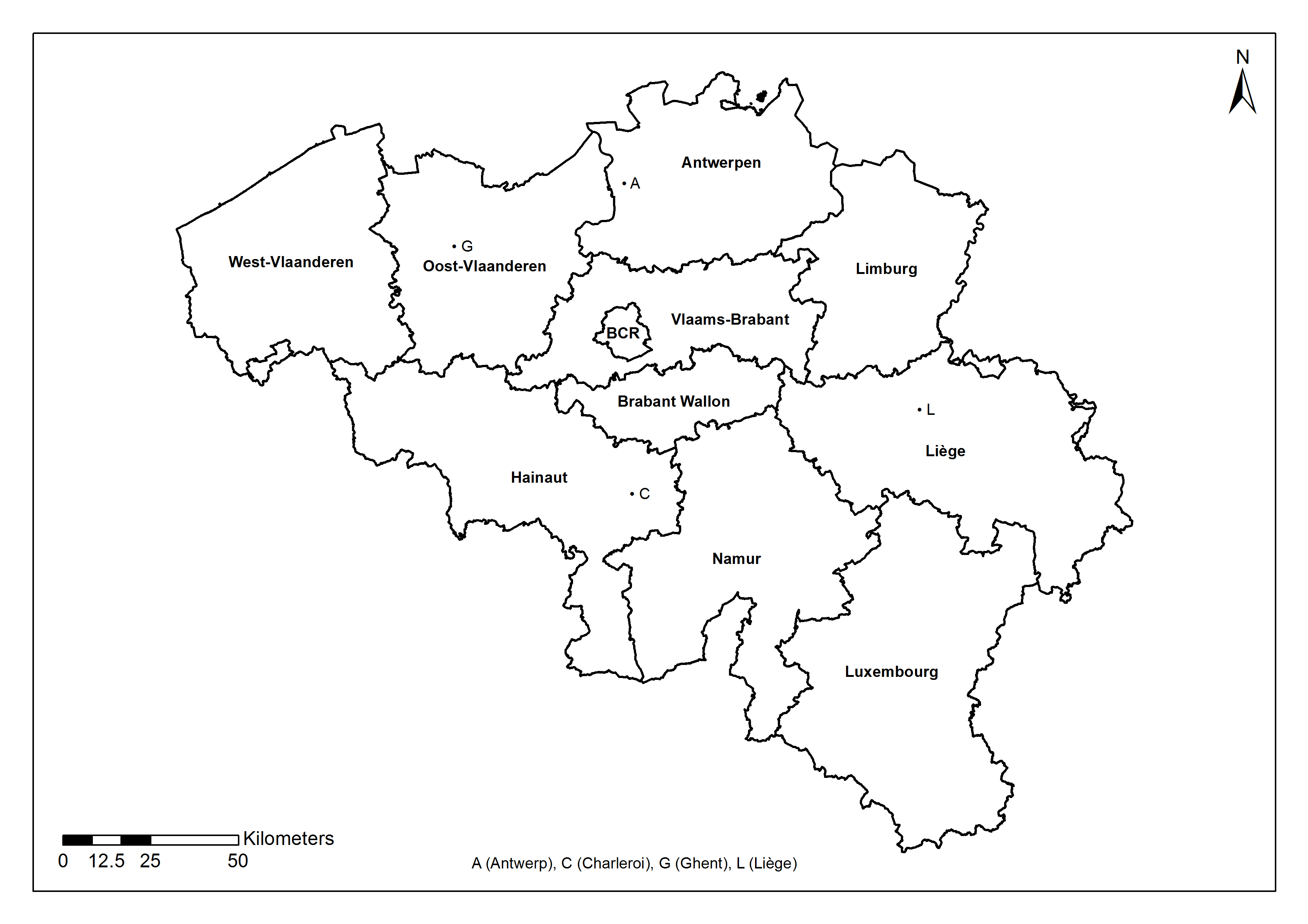

Supplement: Supplementary file 3 — Figure S1. Administrative partition of Belgium. Provinces. West-Vlaanderen, Oost-Vlaanderen, Antwerpen, Vlaams-Brabant (containing the Brussels Capital Region), Limburg, in Flanders; Hainaut, Brabant Wallon, Liège, Namur, Luxembourg, in Wallonia (PNG 139 kb) [file 12931_2019_1052_MOESM3_ESM.png]

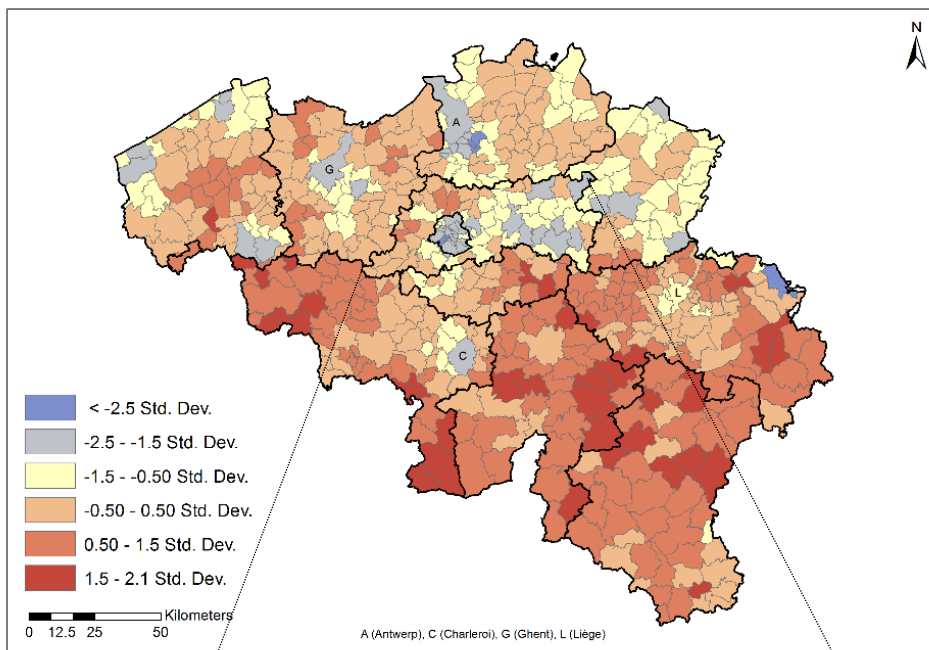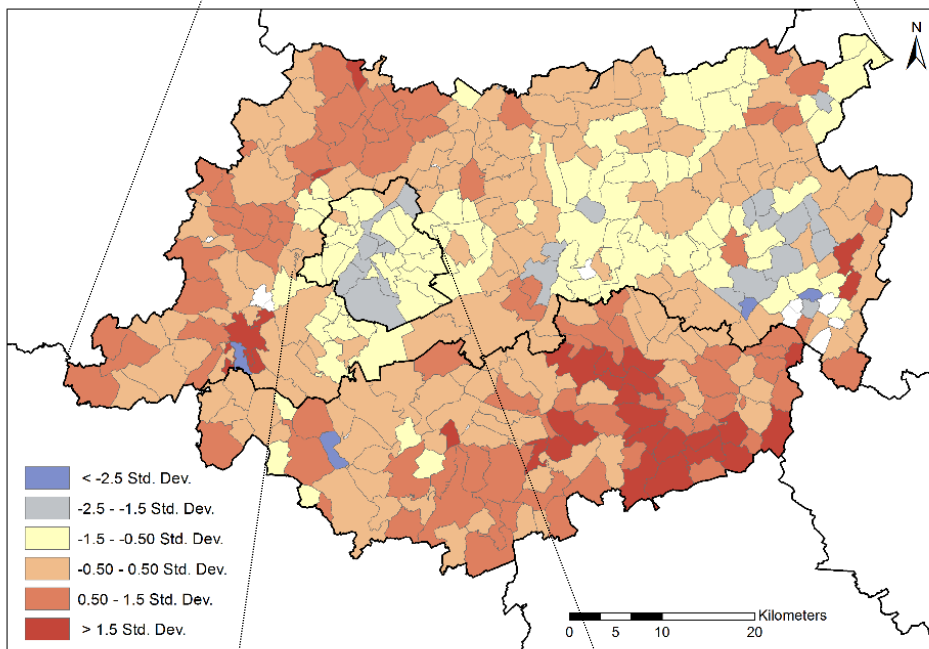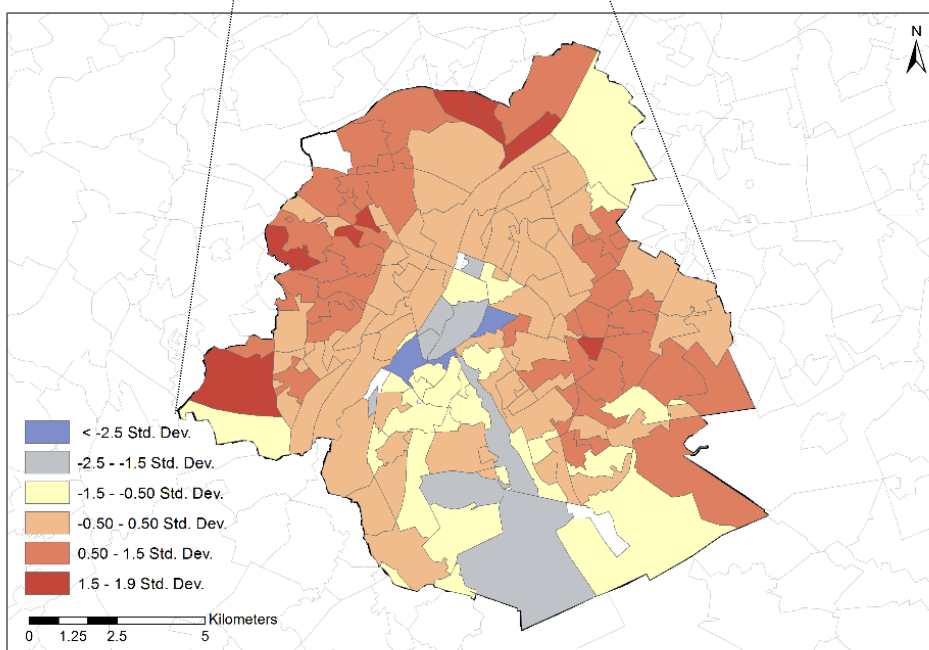

Supplement: Supplementary file 4 — Figure S2. Prevalence of purchase for the three studied areas by means of the discretisation method based on average value and standard deviation (PDF 664 kb) [file 12931_2019_1052_MOESM4_ESM.pdf]
